# What Weight Should Now Be Given to Synovial Fluid Leucocyte Count When Septic Arthritis Is Suspected? A Narrative Review

**DOI:** 10.3390/microorganisms14102189

**Published:** 2026-09-30

**Authors:** Viola Sbampato, Ardian Ramadani, Andreas Tsoupras, Sofia El Hasnaoui, Pierre Antoine Debordes, Giacomo de Marco, Oscar Vazquez, Christina Steiger, Romain Dayer, Dimitri Ceroni

**Affiliations:** Paediatric Orthopaedics Unit, Paediatric Surgery Service, Geneva University Hospitals, CH-1205 Geneva, Switzerlanddimitri.ceroni@hug.ch (D.C.)

**Keywords:** paediatric, septic arthritis, synovial fluid, synovial leucocyte count, synovial fluid analysis, diagnostic threshold

## Abstract

Synovial fluid leucocyte count has long been regarded as a cornerstone in the diagnosis of paediatric septic arthritis, with a threshold of 50,000 cells/mm^3^ widely adopted in clinical practice. This narrative review critically appraised the available evidence regarding its diagnostic utility and interpretation in paediatric septic arthritis. To enhance the transparency and representativeness of the narrative synthesis, rather than to achieve the exhaustive and reproducible study retrieval required for a systematic review, relevant studies were identified through a structured literature search. Fourteen paediatric studies evaluating synovial fluid leucocyte count in the diagnostic assessment of septic arthritis were identified. Whilst several studies reported markedly elevated leucocyte counts in children with septic arthritis, others demonstrated microbiologically confirmed infections below the traditional cut-off, with substantial overlap observed with alternative diagnoses, particularly Lyme arthritis and juvenile idiopathic arthritis. Pathogen characteristics, host inflammatory responses, and prior antibiotic exposure were identified as important factors influencing synovial fluid findings. Current evidence suggests that synovial leucocyte count is best regarded as a marker of intra-articular inflammation rather than a definitive diagnostic test for septic arthritis. Its value lies in contributing to an integrated diagnostic assessment incorporating clinical presentation, imaging findings, microbiological investigations, and emerging molecular diagnostic techniques.

## 1. Introduction

Paediatric septic arthritis is an orthopaedic emergency requiring prompt diagnosis and treatment to prevent irreversible joint damage, growth disturbance, functional impairment, and systemic complications [1]. However, diagnosis remains challenging in children because clinical manifestations are often non-specific, particularly during the early stages of disease, and definitive microbiological confirmation of infection is not immediately available during the initial diagnostic work-up [2,3,4].

To facilitate the early diagnosis of septic arthritis, numerous clinical prediction algorithms have been proposed in the literature, combining clinical findings with serum inflammatory markers [5,6,7]. Nevertheless, synovial fluid analysis remains the cornerstone of the diagnostic evaluation of suspected septic arthritis [8,9,10]. In addition to microbiological investigations, synovial fluid is routinely assessed for its macroscopic appearance, Gram stain, total leucocyte count, and differential cell count, all of which contribute to diagnostic and therapeutic decision-making. Among these parameters, synovial leucocyte count has historically occupied a central role in the diagnostic assessment of septic arthritis. However, growing paediatric evidence has challenged the reliability of interpreting synovial leucocyte count according to a single diagnostic threshold [2,11,12,13,14,15,16,17].

The purpose of this narrative review is to critically evaluate the role of synovial fluid leucocyte count in the diagnosis of acute bacterial septic arthritis in children, hereafter referred to as septic arthritis unless otherwise specified. Specifically, this review examines the biological basis of synovial leucocytosis, the historical development of diagnostic thresholds, contemporary paediatric evidence regarding their diagnostic performance, and the pathogen-, treatment-, and host-related factors that influence synovial leucocyte count, with the aim of defining how this parameter should be interpreted in contemporary paediatric clinical practice.

## 2. Literature Search and Study Selection

This narrative review critically appraised the available evidence regarding the interpretation and diagnostic utility of synovial fluid leucocyte count in paediatric septic arthritis. Relevant studies were identified through a structured literature search.

A structured search of PubMed/MEDLINE, Embase, and the Cochrane Library was conducted from database inception to 31 August 2026. Search terms included combinations of “septic arthritis”, “acute bacterial arthritis”, “paediatric”, “children”, “native joint”, “joint aspiration”, “synovial fluid”, “synovial leucocyte count”, “synovial white blood cell count”, “polymorphonuclear neutrophils”, and “diagnostic threshold”. Reference lists of relevant articles and reviews were also screened to identify additional studies.

Eligible studies included paediatric populations with suspected or confirmed septic arthritis and reported synovial fluid leucocyte counts, diagnostic performance measures, or comparative data from clinically relevant differential diagnoses. Prospective and retrospective cohort studies and case series providing original clinical data were considered. Studies exclusively involving adults, implant- or prosthesis-related joint infections, those not reporting synovial fluid leucocyte counts, and those without relevant diagnostic data were excluded. Non-English publications were excluded unless an English-language version or translation was available through a journal, database, or other reliable academic source. Titles and abstracts of retrieved records were screened for relevance, followed by full-text assessment of potentially eligible articles according to these criteria. Following screening and full-text assessment, fourteen studies met the eligibility criteria and were retained for the narrative synthesis; their main characteristics and findings are summarised in Table 1.

Microbiological confirmation was not required for inclusion, and both microbiologically confirmed and clinically presumed culture-negative cases were considered. Cases in which a bacterial pathogen, including *Kingella kingae*, was identified by pathogen-specific PCR despite negative cultures were considered microbiologically confirmed. Where available, the reference standard and definition of septic arthritis used by each study were recorded and are reported in Table 1. Lyme and brucellar arthritis were considered clinically relevant differential diagnoses rather than primary target conditions, given their distinct clinical and microbiological characteristics and potential overlap in synovial leucocyte counts. Importantly, their epidemiological relevance is strongly geographically dependent, with the pre-test probability of these conditions varying substantially according to regional endemicity and exposure.

As this is a narrative review, the literature search and study-selection process was not designed as a formal systematic or scoping review, and no PRISMA-compliant protocol was applied. The objective was to provide a transparent and representative synthesis of the available paediatric evidence relevant to the interpretation of synovial leucocyte count. Accordingly, no formal risk-of-bias assessment or quantitative evidence synthesis was performed. The heterogeneity of the included studies, particularly with respect to study populations, diagnostic definitions, causative pathogens, joints examined, and reported outcomes, further limited the appropriateness of quantitative pooling.

## 3. Biological Basis of Synovial Leucocytosis

### 3.1. Mechanisms of Leucocyte Recruitment During Joint Infection

Septic arthritis is characterised by the presence of bacteria within the synovial space, followed by activation of a rapid and intense inflammatory response. In children, the synovium is particularly susceptible to haematogenous bacterial invasion because of its rich vascular supply and the absence of a limiting basement membrane, facilitating the translocation of microorganisms into the joint cavity [18,19,20,21]. Once bacteria enter the synovial space, pathogen-associated molecular patterns (PAMPs) are recognised by resident macrophages and synoviocytes through pattern-recognition receptors, including Toll-like receptors, initiating the innate immune response [22,23].

This early inflammatory phase is characterised by the release of pro-inflammatory cytokines, including tumour necrosis factor-α (TNF-α), interleukin (IL)-1β, IL-6, and IL-8 [20,22,24]. These mediators promote endothelial activation, upregulation of adhesion molecules, increased vascular permeability, and leucocyte chemotaxis, thereby facilitating the recruitment of inflammatory cells from the circulation into the infected synovium [22]. Among them, IL-8 (also known as CXCL8) is a potent neutrophil chemoattractant and plays a central role in the recruitment and activation of PMNs within the infected joint [25]. Activated endothelial cells express adhesion molecules that facilitate leucocyte rolling, adhesion, and transmigration into the synovial compartment [22,26,27]. Once within the joint, leucocytes, especially neutrophils, contribute to pathogen clearance through phagocytosis, oxidative burst activity, degranulation, and the formation of neutrophil extracellular traps [28].

Collectively, these coordinated inflammatory events result in rapid synovial leucocytosis, characterised by a predominance of PMNs. Synovial leucocyte count therefore reflects the magnitude of the local inflammatory response, providing the biological basis for its longstanding use as a diagnostic marker of septic arthritis (Figure 1).

### 3.2. Inflammation-Mediated Joint Damage

Beyond their role in pathogen clearance, the inflammatory cells recruited into the synovial compartment contribute directly to articular tissue injury [29]. Activated neutrophils release reactive oxygen species (ROS) and proteolytic enzymes that contribute to bacterial killing but may also cause collateral tissue injury when local antioxidant defences are overwhelmed [30]. In parallel, the inflammatory environment promotes the activation of matrix-degrading enzymes, including matrix metalloproteinases (MMPs), leading to degradation of proteoglycans and collagen within the extracellular matrix (ECM) [31]. Progressive matrix disruption, together with oxidative injury and chondrocyte dysfunction and death, compromises the structural integrity of articular cartilage and may ultimately result in irreversible cartilage loss [32,33]. Importantly, cartilage degradation may begin within the first hours of joint infection, with experimental evidence demonstrating loss of cartilage glycosaminoglycans as early as eight hours after joint infection [34,35].

Articular damage may be further amplified by synovial inflammation and the accumulation of inflammatory exudate within the joint cavity. Increased vascular permeability and cellular infiltration promote synovial oedema and joint effusion, raising intra-articular pressure and potentially compromising local perfusion. Reduced blood supply may contribute to tissue ischaemia and further chondrocyte injury and, particularly in anatomically vulnerable joints such as the shoulder or the hip, may contribute to substantial local morbidity [36]. If infection and inflammation persist, the combined effects of enzymatic degradation, oxidative injury, and impaired perfusion may extend to the subchondral bone and ultimately lead to progressive and potentially irreversible joint destruction [37].

These mechanisms can also be interpreted within the Damage-Response Framework (DRF), which conceptualises disease manifestations and tissue damage as the result of the dynamic interaction between pathogen-associated factors and the host immune response. Within this framework, host damage may arise from microbial virulence factors, an insufficient immune response that permits pathogen persistence, an excessive inflammatory response that itself contributes to tissue injury, or a combination of these mechanisms [38]. This interplay is particularly relevant in septic arthritis, in which immune activation is essential for pathogen control but may simultaneously contribute to articular damage. Collectively, these mechanisms demonstrate that synovial leucocytosis is not merely a diagnostic finding but also reflects an intra-articular inflammatory response capable of contributing to progressive articular damage.

### 3.3. Factors Influencing Synovial Leucocyte Count

Although synovial leucocyte count reflects the intensity of intra-articular inflammation, its magnitude varies considerably between patients owing to a complex interplay of pathogen-, treatment-, and host-related factors (Figure 2). These factors are frequently interrelated; for example, age influences the epidemiological distribution of causative pathogens in paediatric septic arthritis and may therefore indirectly affect the magnitude of the synovial inflammatory response. This heterogeneity helps explain why no single synovial leucocyte threshold can reliably distinguish septic arthritis from alternative diagnoses.

One of the principal determinants of synovial fluid cellularity is the causative pathogen. Infections caused by highly pyogenic organisms, such as *S. aureus*, are generally associated with marked neutrophilic inflammation and substantial synovial leucocytosis. In contrast, *K. kingae*, now recognised as a leading cause of osteoarticular infection in children younger than four years, often produces a milder clinical and biological inflammatory response [12]. Several studies have reported relatively modest synovial leucocyte counts in children with *K. kingae* septic arthritis, consistent with its generally less pronounced inflammatory phenotype [2,18,39]. However, exceptions have been reported. Basmaci et al. reported a higher median synovial leucocyte count in children with *K. kingae* arthritis than in those with *S. aureus* arthritis, despite the milder clinical presentation associated with *K. kingae* [11]. Consequently, differences in synovial leucocyte count between paediatric cohorts may partly reflect differences in age distribution and the associated pathogen spectrum, rather than differences in the intrinsic diagnostic performance of synovial leucocyte count itself.

*K. kingae* possesses several virulence and immune-evasion mechanisms that may contribute to its comparatively attenuated inflammatory phenotype. Type IV pili mediate initial adherence to host cells, while subsequent pilus retraction facilitates Knh-mediated close attachment [40]. The polysaccharide capsule and galactan exopolysaccharide promote immune evasion by reducing neutrophil recognition, phagocytosis, and killing and protecting against complement-mediated lysis [41]. In addition, the RtxA toxin exerts cytotoxic effects on epithelial, synovial, and macrophage-like cells and may contribute to immune evasion through cytotoxicity towards recruited phagocytic cells [42]. *K. kingae* also carries the phase-variable Type III DNA methyltransferase ModK; ModK1 “ON” variants have been associated with reduced macrophage production of pro-inflammatory cytokines, including IL-8, IL-1β, and TNF [43]. Collectively, these mechanisms may facilitate persistence while attenuating host inflammatory responses, providing a plausible biological basis for the comparatively mild inflammatory phenotype observed in *K. kingae* infection [40,41,42,43].

Treatment-related factors, particularly antibiotic administration before arthrocentesis, may also contribute to variability in synovial fluid findings. Empirical antimicrobial therapy initiated before joint aspiration may attenuate the local inflammatory response and alter synovial fluid findings [44]. However, much of the evidence supporting this effect derives from adult populations and should therefore be regarded as indirect evidence when considering paediatric septic arthritis. Puzzitiello et al. reported lower synovial leucocyte counts following pre-aspiration antibiotic administration in adults with native-joint septic arthritis, while Massey et al. similarly demonstrated that the optimal diagnostic leucocyte threshold was lower in a predominantly adult cohort receiving antibiotics before arthrocentesis [44,45].

Paediatric-specific evidence remains considerably more limited. Obana et al. found no significant association between prior antibiotic exposure and synovial leucocyte count or PMN proportion in children with septic arthritis [10]. Other paediatric studies have included relatively small numbers of pretreated patients or have not reported antibiotic exposure in sufficient detail to permit robust conclusions [46,47]. Interpretation of these apparently conflicting findings is limited by the inconsistent reporting of antibiotic exposure across studies, particularly regarding the antimicrobial agent administered, duration of treatment, and interval between antibiotic administration and joint aspiration. Differences in these factors, together with variation in study populations and sample sizes, may contribute to the observed discrepancies. Future paediatric studies should therefore report pre-aspiration antibiotic exposure in a standardised manner, including the agent administered, duration of therapy, and interval between administration and synovial fluid collection. Whenever clinically feasible, synovial fluid should be obtained before the initiation of antimicrobial therapy.

Host-related factors also contribute to variability in synovial fluid findings through differences in the inflammatory response. Immune status and underlying inflammatory disorders may modulate this response [48,49] such that synovial leucocyte counts may vary even among patients with the same infection. Moreover, because intense intra-articular inflammation is not specific to bacterial infection, conditions such as Lyme arthritis and juvenile idiopathic arthritis may also produce marked synovial leucocytosis despite the absence of pyogenic pathogens [50,51]. These observations reinforce that synovial leucocyte count primarily reflects the magnitude of the local inflammatory response rather than the presence of bacterial infection itself.

## 4. Historical Development of Diagnostic Thresholds

Historically, interpretation of synovial fluid in suspected septic arthritis has relied heavily on numerical thresholds intended to distinguish bacterial infection from non-infectious inflammatory joint disease. Among these, a synovial leucocyte count exceeding 50,000 cells/mm^3^ [45,52,53,54,55] and a PMN proportion greater than 90% became widely used indicators of septic arthritis [56,57,58].

These cut-offs were largely informed by studies demonstrating higher synovial leucocyte counts and neutrophil proportions in septic arthritis than in non-infectious joint conditions. As readily available parameters in the absence of immediate microbiological confirmation, they became incorporated into the diagnostic assessment of suspected septic arthritis.

However, the evidence underlying these conventional thresholds was derived predominantly from adult or mixed-age populations, with limited validation in children. Their diagnostic applicability to paediatric septic arthritis therefore requires consideration in the context of contemporary paediatric evidence.

**Table 1:** Summary of paediatric studies assessing the diagnostic utility of synovial leucocyte count in septic arthritis.

Study	Population	Reference Standard	Pathogen/Joint	Synovial WBC Findings (cells/mm^3^)	Key Interpretation
Press et al. 2002 [13]	9 children; median age 7 years	Positive synovial fluid culture	*B. melitensis*; mainly knee, hip and shoulder	Median WBC: 9500 (range 300–61,500)	Most culture-confirmed cases had WBC <50,000 cells/mm^3^ (8/9; 88.9%), demonstrating that values below this threshold do not exclude infection.
Basmaci et al. 2011 [11]	90 children; *K. kingae* SA n = 64; *S. aureus* SA n = 26	Positive synovial fluid culture or molecular diagnosis	*K. kingae*/*S. aureus*; mainly knee and hip	Median WBC: *K. kingae* 137,000 (range 5900–3,200,000; n = 33); *S. aureus* 48,000 (range 200–730,000; n = 12)	Microbiologically confirmed SA showed wide WBC variability above and below 50,000 cells/mm^3^, limiting the reliability of this threshold for predicting SA.
Williams et al. 2014 [12]	27 children; median age 18.6 months	*K. kingae* PCR-positive synovial fluid	*K. kingae*; knee 63% (n = 17), ankle 18.5% (n = 5), hip 7.4% (n = 2), shoulder 7.4% (n = 2), elbow 3.7% (n = 1)	Median WBC: 21,800 (IQR 16,700–45,000; n = 11)	PCR-confirmed *K. kingae* SA may present with WBC <50,000 cells/mm^3^, demonstrating that this threshold may miss infection.
Heyworth et al. 2015 [14]	46 children; mean age 7.6 years; SA n = 15 (33%); Lyme arthritis n = 13 (28%); transient synovitis n = 8 (17%); other diagnoses n = 10 (22%)	SA: positive bacterial culture or culture-negative SA based on clinical consensus and retrospective author review; Lyme arthritis: positive Western blot and/or synovial fluid PCR	Hip; *S. aureus* was the identified pathogen in culture-positive SA	Mean WBC: SA 55,700 (n = 15); Lyme arthritis 53,500 (n = 13); transient synovitis 41,500 (n = 8); other diagnoses 42,300 (n = 10)	A WBC ≥50,000 cells/mm^3^ increased the probability of SA (48% [11/23] vs. 17% [4/23] below the threshold; OR 4.4, 95% CI 1.1–16.9; *p* = 0.03), but SA remained present below this threshold, limiting the reliability of 50,000 cells/mm^3^ as an isolated diagnostic cut-off.
Baldwin et al. 2016 [59]	189 children; culture-positive SA n = 23; culture-negative SA n = 26; Lyme arthritis n = 140	Culture-positive SA: positive synovial fluid culture; culture-negative SA: WBC >60,000 cells/mm^3^ + negative Lyme titre; Lyme arthritis: positive serum IgG Western blot	Knee; culture-positive SA predominantly *S. aureus*; Lyme arthritis: B. burgdorferi	Mean WBC: culture-positive SA 128,100; Lyme arthritis 53,600	Although WBC was higher in culture-positive SA, Lyme arthritis frequently produced values ≥50,000 cells/mm^3^, limiting the specificity of this threshold for predicting SA.
Aupiais et al. 2017 [3]	133 children overall; median age 2.2 years; 125 included in the main comparison: SA n = 63; no definitive diagnosis n = 45; JIA n = 17; other diagnoses n = 6; unclassifiable n = 2	SA: microbiologically confirmed by blood or synovial fluid culture or molecular methods (*K. kingae*-specific real-time PCR or universal 16S rRNA PCR)	Mixed; *K. kingae* 79.4%; *S. aureus* 7.9%; knee 57.2%; hip 23.8%; ankle 9.5%; other 9.5%	Median WBC: SA 147,000; no definitive diagnosis 10,000; JIA 51,000	A WBC >50,000 cells/mm^3^ was present in 86.2% of SA cases (n = 25); however, 50% of JIA cases with available synovial WBC data (n = 4) also exceeded this threshold, demonstrating substantial overlap between infectious and inflammatory arthritis.
Spyridakis et al. 2019 [15]	157 children; SA without OM n = 129 (culture-positive n = 40; culture-negative n = 89); SA with OM n = 28	SA: positive joint or blood culture; culture-negative SA: clinical symptoms + WBC >50,000 cells/mm^3^ + clinical diagnosis	Mixed; predominantly *S. aureus*; hip and knee most common	Median WBC: culture-positive SA 110,000 (IQR 56,400–212,100); culture-negative SA 115,000 (IQR 82,500–180,000)	Similar WBC values were observed in culture-positive and culture-negative SA; however, incorporation of WBC >50,000 cells/mm^3^ into the culture-negative SA definition prevents independent assessment of the predictive value of this threshold.
Illán-Ramos et al. 2018 [60]	5 children; median age 24 months	*K. kingae* identified by 16S rRNA PCR/sequencing of synovial fluid	*K. kingae*; knee n = 4; hip n = 1	Median WBC: 61,322 (range 10,040–78,320; n = 4)	Molecularly confirmed SA occurred with WBC <50,000 cells/mm^3^ in 25% (1/4) of cases with available WBC, demonstrating that this threshold may miss infection
Dart et al. 2018 [47]	238 children; median age 6 years; SA n = 26 (11%); Lyme arthritis n = 32 (13%); other inflammatory arthritis n = 180 (76%)	SA: positive synovial fluid culture or WBC ≥50,000 cells/mm^3^ + positive blood culture; Lyme arthritis: positive two-tier serology + negative synovial fluid culture	Hip; SA: *S. aureus* n = 24, *K. kingae* n = 2; Lyme arthritis: *B. burgdorferi*	Median WBC: SA 126,100 (IQR 83,300–209,300); Lyme arthritis 54,000 (IQR 33,800–73,400); other inflammatory arthritis 20,300	A WBC ≥50,000 cells/mm^3^ identified 81% of SA cases, but 19% of SA occurred below this threshold and 56% of Lyme arthritis cases (18/32) exceeded it, limiting its reliability for predicting SA.
Obana et al. 2019 [10]	166 patients undergoing 172 joint aspirations; mean age 7.9 years; C-SA n = 44; S-SA n = 45; Other n = 83 (including Other-R n = 21)	C-SA: positive synovial fluid culture; S-SA: negative culture with discharge diagnosis of SA or SA with adjacent OM and operative treatment	Mixed pathogens; Knee 55% (n = 95); hip 29% (n = 50); other joints 16% (n = 27)	Mean WBC: C-SA 92,000; S-SA 54,000; other 10,000.	Although mean WBC was significantly higher in C-SA and S-SA than in the Other group, the wide variability and overlap between groups limit the reliability of a fixed 50,000 cells/mm^3^ threshold for diagnosing SA.
Thomas et al. 2021 [50]	122 children; SA n = 88 (true SA n = 51; presumed SA n = 37); aseptic effusion n = 34	True SA: positive joint culture or WBC >50,000 cells/mm^3^ + positive blood culture; presumed SA: negative cultures with WBC >50,000 cells/mm^3^, or WBC >25,000 cells/mm^3^ + clinical diagnosis	Mixed pathogens; knee	Median WBC: SA 79,050 (IQR 47,343–127,118); aseptic effusion 16,242 (IQR 6674–37,485). Among true SA cases with WBC <50,000: mean WBC 22,000 (range 839–40,250)	A WBC <50,000 cells/mm^3^ occurred in 17.6% (9/51) of true SA cases, demonstrating that this threshold may miss infection; however, incorporation of WBC into the case definitions limits independent assessment of its predictive value.
Otsubo et al. 2024 [16]	22 children; median age 5 years	Positive synovial fluid culture	Mixed; MSSA 50%; *S. pyogenes* 27%; MRSA 9%; other pathogens 15%; hip 59%; knee 23%; other joints 18%	Median WBC: 19,575 (IQR 6806–47,388); Mean WBC: 31,977	Most culture-confirmed SA cases had WBC <50,000 cells/mm^3^ (17/22; 77.3%), demonstrating that this threshold may miss a substantial proportion of infections
Nyaaba et al. 2022 [46]	30 children; median age 43 months; microbiologically confirmed SA n = 17; controls n = 13	SA: pathogen detected in synovial fluid by culture or molecular testing	Knee; *K. kingae* n = 9; *S. aureus* n = 4; *S. pyogenes* n = 2; *E. coli* n = 1; *S. mitis* n = 1	Median WBC: SA 70,400; controls 5000; AUC 0.86 (95% CI 0.73–1.00)	Although synovial WBC was significantly higher in microbiologically confirmed SA, no reliable minimum threshold, including 50,000 cells/mm^3^, could be established for predicting SA.
Dechnik et al. 2024 [17]	796 children; culture-positive SA n = 79 (10%)	Positive synovial fluid culture	Mixed pathogens; multiple joints	WBC ≥50,000 cells/mm^3^: sensitivity 62.0% (95% CI 50.4–72.7%); specificity 67.0% (95% CI 63.4–70.4%); AUC 0.72 (95% CI 0.65–0.78)	A WBC ≥50,000 cells/mm^3^ had a sensitivity of 62% and specificity of 67%; approximately 30/79 (38%) culture-positive SA cases therefore fell below this threshold, demonstrating its limited ability to exclude SA.

Abbreviations: C-SA, confirmed septic arthritis; IQR, interquartile range; JIA, juvenile idiopathic arthritis; MRSA, methicillin-resistant Staphylococcus aureus; MSSA, methicillin-susceptible Staphylococcus aureus; OM, osteomyelitis; PCR, polymerase chain reaction; rRNA, ribosomal RNA; SA, septic arthritis; S-SA, suspected septic arthritis; WBC, white blood cell count.

## 5. Contemporary Paediatric Evidence

Recent paediatric studies have challenged the diagnostic reliability of the traditional synovial leucocyte threshold of 50,000 cells/mm^3^ for septic arthritis. As summarised in Table 1, higher synovial leucocyte counts are generally associated with bacterial infection; however, microbiologically confirmed infections may occur below this cut-off, while inflammatory and other infectious arthritides may produce values above it [3,5,10].

Several paediatric studies have documented septic arthritis despite synovial leucocyte counts below 50,000 cells/mm^3^. Williams et al. reported a median synovial leucocyte count of 21,800 cells/mm^3^ in children with PCR-confirmed *K. kingae* septic arthritis [12]. Low counts have also been reported in other infectious arthritides within the differential diagnosis; Press et al. similarly found a median count of 9500 cells/mm^3^ in children with microbiologically confirmed brucellar arthritis [13]. Thomas et al. found that 9 of 51 (17.6%) children with true septic arthritis had synovial leucocyte counts below 50,000 cells/mm^3^, with a mean count of 22,000 cells/mm^3^ in this subgroup [50]. In the diagnostic ‘grey zone’ examined by Heyworth et al., septic arthritis was diagnosed in 17% of children with synovial leucocyte counts below 50,000 cells/mm^3^, compared with 48% of those with counts ≥50,000 cells/mm^3^. Although a count ≥50,000 cells/mm^3^ was associated with significantly higher odds of septic arthritis, these findings demonstrate that increasing leucocytosis raises the probability of septic arthritis without providing an absolute diagnostic boundary [2,14,50,61].

This variability persists even in cohorts with generally high synovial leucocyte counts. Dart et al. observed a median count of 126,130 cells/mm^3^ in septic arthritis, yet 19% of confirmed cases had values below 50,000 cells/mm^3^ [47]. Similarly, Illán-Ramos et al. reported a median of 61,322 cells/mm^3^ in *K. kingae* septic arthritis, while one of four patients with available synovial leucocyte counts (25%) had a value below the traditional cut-off [60].

Conversely, values above 50,000 cells/mm^3^ are not specific for pyogenic infection. Baldwin et al. reported a mean synovial leucocyte count of 53,640 cells/mm^3^ in children with Lyme arthritis, exceeding the conventional threshold [59]. Similarly, Dart et al. found that 56% of children with Lyme arthritis had counts >50,000 cells/mm^3^ [47]. Comparable overlap has been observed in inflammatory rheumatological disease, with Aupiais et al. reporting values above 50,000 cells/mm^3^ in approximately half of children with juvenile idiopathic arthritis [3].

Interpretation of these findings is also influenced by differences in the reference standards used across studies. Some studies defined septic arthritis exclusively by microbiological confirmation, whereas others included clinically presumed culture-negative cases or incorporated synovial leucocyte count itself into the diagnostic definition. The latter introduces incorporation bias, because the index test forms part of the reference standard against which its diagnostic performance is evaluated, potentially inflating its apparent diagnostic accuracy. Conversely, reliance on microbiological confirmation alone may misclassify true culture-negative infections, particularly following prior antibiotic exposure or in infections caused by fastidious organisms. Such differences in reference standards may therefore contribute to the heterogeneity in reported diagnostic performance and limit direct comparison of synovial leucocyte thresholds across studies.

Taken together, these findings support interpreting synovial leucocyte count as a continuous diagnostic variable rather than a binary test. Increasing synovial leucocytosis is associated with a greater likelihood of septic arthritis, but no single threshold reliably confirms or excludes the diagnosis in children.

## 6. Clinical Implications and Future Perspectives

From a clinical perspective, synovial leucocyte count should no longer be interpreted as an isolated diagnostic criterion but rather as one component of an integrated and probabilistic diagnostic assessment. Its diagnostic significance depends on the pre-test probability of acute bacterial arthritis, which is determined by the clinical presentation, age, epidemiological context, serum inflammatory markers, imaging findings, and the likelihood of specific pathogens. The synovial leucocyte count should therefore be interpreted as a continuous variable that modifies this initial probability rather than as a binary test that independently confirms or excludes infection.

Accordingly, the same synovial leucocyte count may have different diagnostic implications depending on the clinical context. For example, a count of 40,000 cells/mm^3^ should not substantially reduce concern for acute bacterial arthritis in a febrile, systemically unwell child who refuses weight bearing and has markedly elevated inflammatory markers. Conversely, the same value in an otherwise well child presenting with isolated knee swelling in a Lyme-endemic region may carry a different diagnostic weight. Conceptually, the diagnostic process can therefore be considered as a sequence from pre-test probability, through interpretation of the synovial leucocyte count and PMN proportion, to a revised post-test probability, which is subsequently refined by microbiological, clinical, and imaging findings (Figure 3).

This approach is broadly consistent with the 2023 PIDS/IDSA guideline for acute bacterial arthritis in children, which recommends synovial leucocyte count and differential as part of the initial evaluation while emphasising that these findings should be interpreted alongside the clinical presentation and other laboratory findings [9]. The guideline also supports molecular testing when routine microbiological investigations fail to identify a pathogen, particularly in preschool-aged children at risk of *K. kingae* infection. Decisions regarding joint drainage are likewise based on the overall clinical context, including the severity of infection, the affected joint, and the gross appearance and cellular profile of the synovial fluid, rather than on a single synovial leucocyte threshold [9]. The present review therefore largely aligns with current guideline recommendations while further emphasising synovial leucocyte count as a continuous, probability-modifying variable rather than a fixed diagnostic threshold.

As molecular diagnostic techniques continue to improve pathogen detection, future diagnostic strategies are likely to rely increasingly on this integrated approach. Conventional Gram staining and culture remain fundamental, but the increasing availability of molecular diagnostic techniques has substantially expanded the microbiological investigation of culture-negative infections.

Since the early 2000s, the increasing use of nucleic acid amplification tests (NAATs) has substantially improved the microbiological diagnosis of paediatric osteoarticular infections and has contributed to a better understanding of their bacterial epidemiology. Targeted PCR-based testing represents an important advance by allowing the rapid detection of pathogen-specific nucleic acid directly in clinical specimens, including in infections caused by fastidious organisms that may remain undetected by conventional culture [62]. Advances in NAAT technology, particularly the development of species-specific real-time PCR assays, have improved diagnostic performance while reducing some of the limitations associated with broad-range amplification techniques.

This evolution is particularly relevant to *K. kingae*, which is now recognised as a leading cause of osteoarticular infection in children younger than four years [63]. Species-specific PCR has substantially increased its detection, including in culture-negative cases, and has consequently revealed a larger population of microbiologically confirmed infections associated with a comparatively mild clinical and biological inflammatory response [64]. Importantly, however, more than 20% of paediatric osteoarticular infections may remain microbiologically undocumented despite contemporary molecular testing, suggesting that additional fastidious or as-yet unrecognised organisms may remain undetected with currently available targeted assays [65,66]. Molecular diagnostic advances have also improved the identification of other infectious arthritides, including those caused by *Borrelia burgdorferi* and *Brucella* spp., although these remain clinically relevant differential diagnoses rather than the primary diagnostic target of this review [67,68].

These developments may have important implications for the interpretation of synovial leucocyte count. The increasing use of targeted molecular assays allows microbiological confirmation of infections that might previously have remained culture-negative, microbiologically undocumented, or diagnostically uncertain, including infections caused by less pyogenic organisms [62,63,64,65,66]. Such cases may present with less pronounced synovial leucocytosis and may therefore partly explain why contemporary evidence increasingly reports microbiologically confirmed infections below the traditional threshold of 50,000 cells/mm^3^. Thus, the increasing recognition of septic arthritis presenting with lower synovial leucocyte counts should not necessarily be interpreted as a change in the biological behaviour of the disease, but may partly reflect improved microbiological detection across a broader spectrum of pathogen virulence, bacterial burden, and disease stage.

Rather than replacing synovial fluid analysis, these techniques should be regarded as complementary investigations that further refine the probability of infection, particularly in diagnostically challenging cases.

Additional biochemical parameters may also provide complementary information when interpreted alongside synovial leucocyte count. Changes in synovial glucose, lactate, and total protein have been investigated as potential indicators of bacterial joint infection, although their diagnostic performance and clinical utility remain insufficiently established, particularly in children [69,70]. Procalcitonin has also emerged as a potential biomarker of bacterial arthritis. Aggarwal et al. reported significantly higher serum and synovial procalcitonin concentrations in patients with pyogenic compared with non-pyogenic arthritis, with synovial procalcitonin demonstrating slightly greater diagnostic accuracy than serum procalcitonin (AUC 0.914 vs. 0.895) [71]. However, the available evidence remains limited and is predominantly derived from adult or mixed-age populations. These parameters should therefore currently be regarded as complementary rather than standalone diagnostic markers, and further paediatric-specific studies are required.

Imaging should similarly be interpreted as a complementary component of this diagnostic framework rather than as an independent discriminator of infection or a substitute for synovial fluid analysis [72,73]. Conventional MRI can contribute to the differentiation of septic from non-septic arthritis through features such as synovial enhancement, periarticular soft-tissue abnormalities, and bone marrow changes; however, joint effusion itself remains non-specific, and substantial overlap exists between septic and reactive effusions [72]. Emerging techniques, including DWI and T2-relaxometry, may provide additional information on joint-fluid characteristics, but their role in paediatric septic arthritis remains investigational [74,75]. Whether these approaches provide diagnostic value beyond established clinical, synovial, and microbiological parameters requires further investigation.

Future studies should prospectively evaluate synovial leucocyte count in large, multicentre paediatric cohorts using standardised definitions of acute bacterial arthritis and systematic microbiological testing. Rather than seeking another universal cut-off, future research should assess synovial leucocyte count as a continuous variable and determine its incremental diagnostic value when combined with clinical findings, serum inflammatory markers, PMN proportion, culture, and molecular diagnostics. Future studies should also account for the diagnostic method used for pathogen identification, as increasing use of targeted molecular assays may alter the spectrum of microbiologically confirmed disease and, consequently, the observed distribution of synovial leucocyte counts. Stratification by age, causative pathogen, joint involved, and prior antibiotic exposure would further clarify the factors influencing its diagnostic performance.

## 7. Conclusions

Synovial fluid leucocyte count remains an important component of the diagnostic evaluation of children with suspected septic arthritis. Although increasing synovial leucocytosis is associated with a greater probability of bacterial infection, contemporary paediatric evidence demonstrates that no single synovial leucocyte threshold can reliably confirm or exclude the diagnosis.

Rather than representing an absolute diagnostic criterion, synovial leucocyte count should be regarded as a marker of intra-articular inflammation. In clinical practice, a value below 50,000 cells/mm^3^ should not exclude septic arthritis when clinical suspicion remains high, while a value above this threshold should not be considered diagnostic of bacterial infection in isolation. Its interpretation must therefore be integrated with the clinical presentation, serum inflammatory markers, imaging findings, microbiological investigations, and, increasingly, molecular diagnostic techniques.

## Figures and Tables

**Figure 1:**
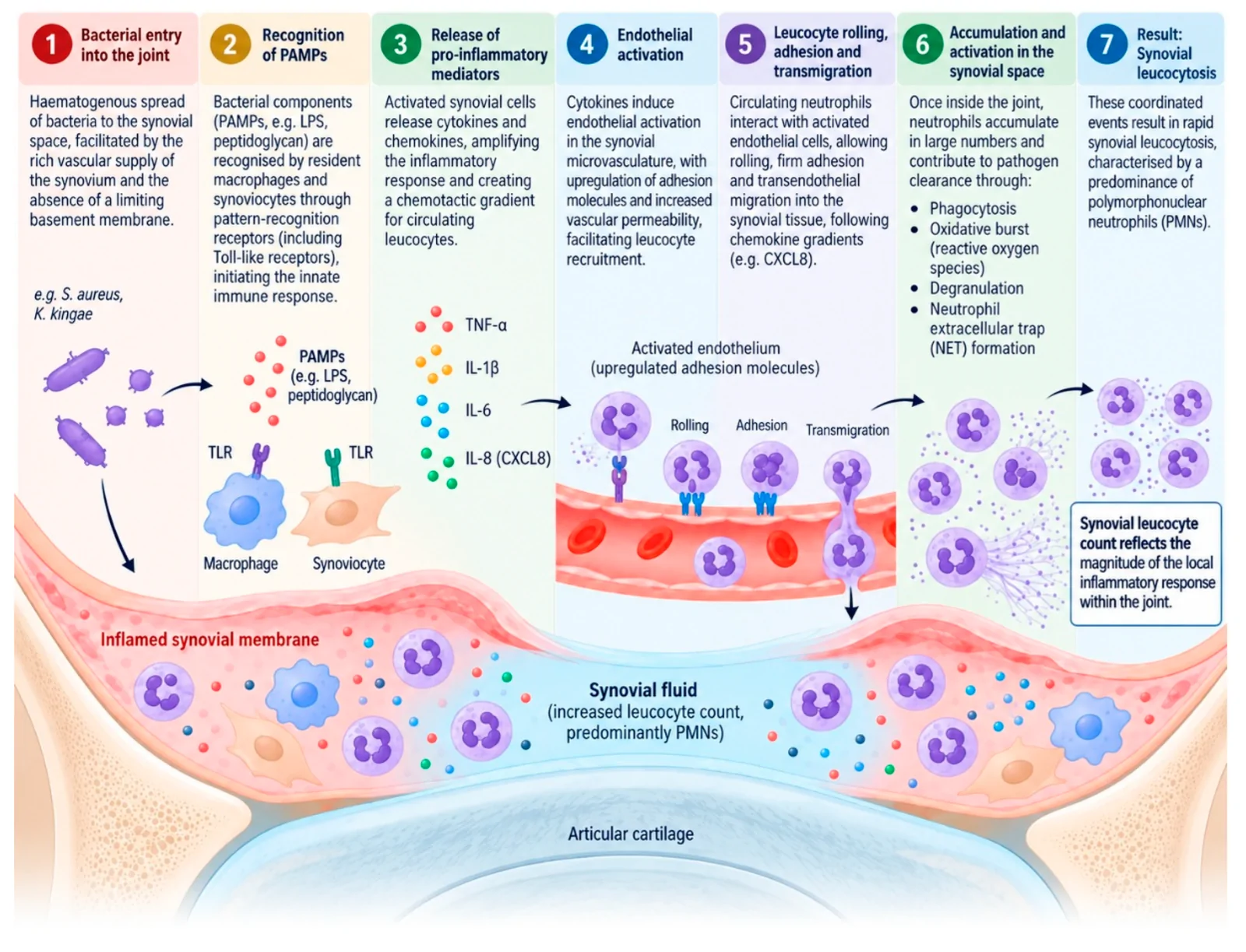
Mechanism of leucocyte recruitment during joint infection. Bacterial entry and recognition trigger pro-inflammatory signalling, endothelial activation, and neutrophil recruitment into the synovial space, resulting in PMN-predominant synovial leucocytosis. Coloured dots represent the different inflammatory mediators indicated in the figure (TNF-α, IL-1β, IL-6, and IL-8/CXCL8).

**Figure 2:**
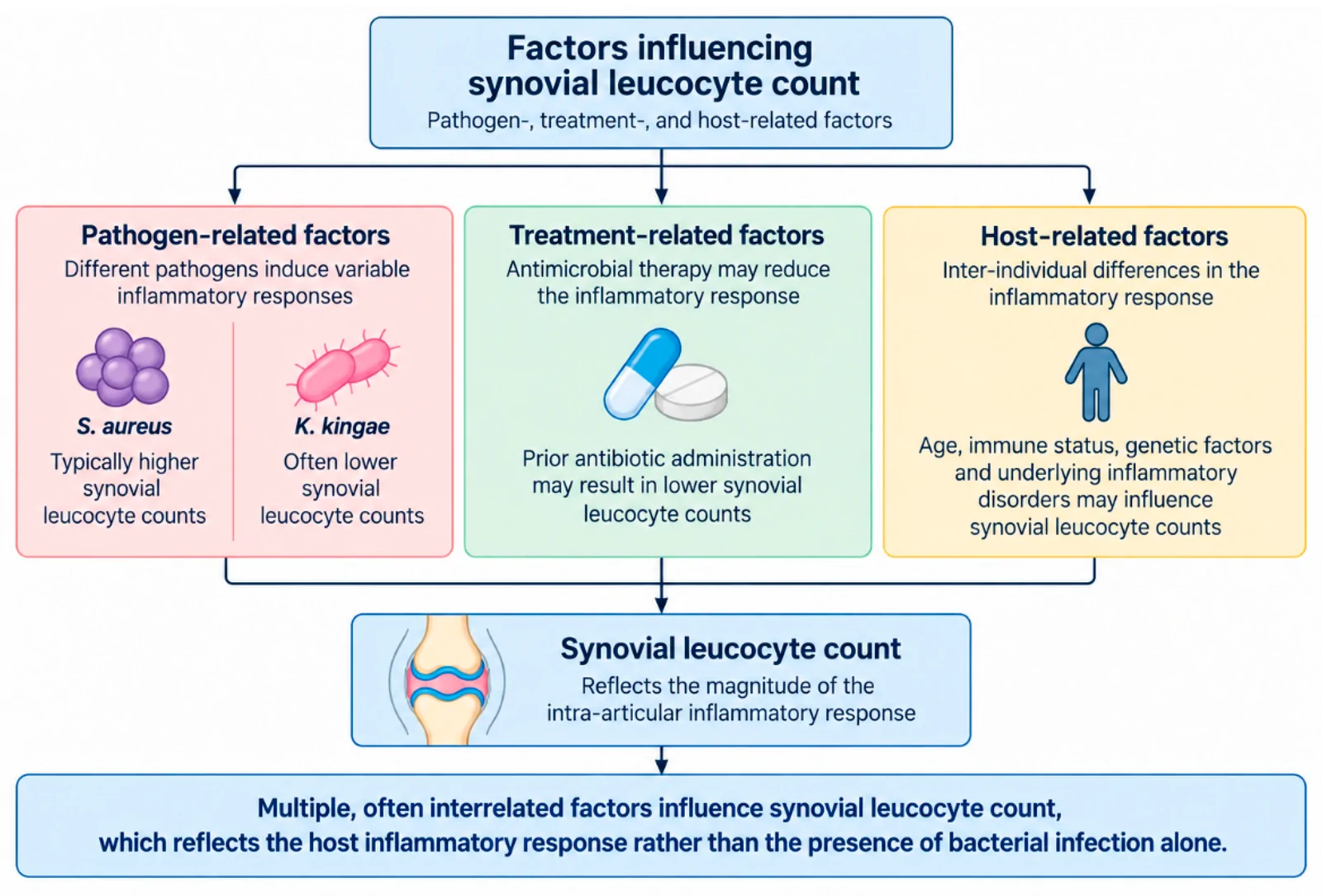
Factors influencing synovial leucocyte count. Synovial leucocyte count is influenced by a complex interplay of pathogen-, treatment-, and host-related factors. These factors contribute to substantial variability in synovial leucocytosis, reinforcing its interpretation as a marker of the magnitude of intra-articular inflammation rather than of bacterial infection alone.

**Figure 3:**
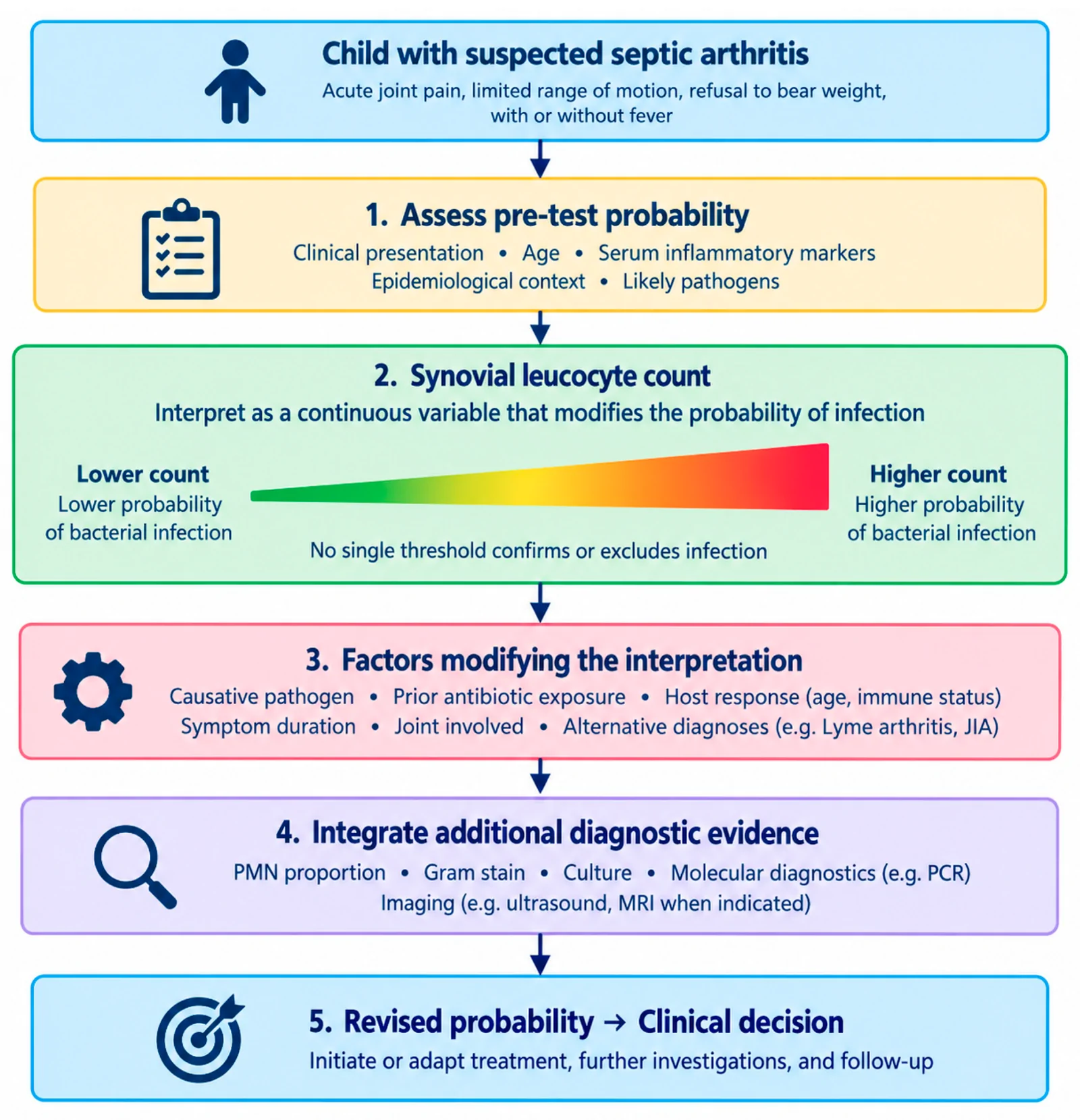
Integrated diagnostic interpretation of synovial leucocyte count in suspected paediatric septic arthritis. Synovial leucocyte count should be interpreted as a continuous variable that modifies the pre-test probability of infection. Its interpretation should integrate clinical context and additional diagnostic evidence to refine the probability of infection and guide clinical decision-making.

## Data Availability

No new data were created or analysed in this study. Data sharing is not applicable to this article.

## Abbreviations

The following abbreviations are used in this manuscript:

AUC	Area under the curve
C-SA	Confirmed septic arthritis
CXCL8	C-X-C motif chemokine ligand 8
DRF	Damage-Response Framework
DWI	Diffusion-weighted imaging
ECM	Extracellular matrix
IL	Interleukin
IL-1β	Interleukin-1 beta
IL-6	Interleukin-6
IL-8	Interleukin-8
IQR	Interquartile range
JIA	Juvenile idiopathic arthritis
*K. kingae*	*Kingella kingae*
MMPs	Matrix metalloproteinases
MRI	Magnetic resonance imaging
MRSA	Methicillin-resistant *Staphylococcus aureus*
MSSA	Methicillin-susceptible *Staphylococcus aureus*
NAATs	Nucleic acid amplification tests
OM	Osteomyelitis
PAMPs	Pathogen-associated molecular patterns
PCR	Polymerase chain reaction
PMN/PMNs	Polymorphonuclear neutrophil(s)
ROS	Reactive oxygen species
rRNA	Ribosomal RNA
*S. aureus*	*Staphylococcus aureus*
SA	Septic arthritis
S-SA	Suspected septic arthritis
TNF-α	Tumour necrosis factor alpha
WBC	White blood cell
